# *Schiedeella bajaverapacensis* (Orchidaceae, Spiranthinae), a New Orchid Species from Guatemala

**DOI:** 10.3390/ijms24065362

**Published:** 2023-03-10

**Authors:** Fredy L. Archila Morales, Monika M. Lipińska, Magdalena Dudek, Dariusz L. Szlachetko

**Affiliations:** 1 Estación Experimental de Orquídeas de la Familia Archila, Cobán 16001, Guatemala; 2 Herbario BIGU, Escuela de Biología, Facultad de Ciencias Químicas y Farmacia, Universidad de San Carlos de Guatemala, Guatemala City 01012, Guatemala; 3 Department of Plant Taxonomy and Nature Conservation, Faculty of Biology, University of Gdańsk, 80-308 Gdansk, Poland; 4 Foundation Polish Orchid Association, 81-825 Sopot, Poland

**Keywords:** Central America, new species, orchids, phylogeny, *Spiranthes*, taxonomy

## Abstract

Guatemala is recognized for its diverse and rich flora and fauna. It is estimated that over 1200 orchid species, classified in 223 genera, are known to occur in this rather small, yet megadiverse country. While studying the diversity of this plant group in the department of Baja Verapaz, we found individuals that clearly belonged to the genus *Schiedeella*, but whose features did not fit any previously known species. At that time, nine terrestrial taxon representatives were known to occur in Guatemala. We conducted the morphological analysis in accordance with the standard procedures of classical taxonomy. For phylogenetic reconstruction, 59 sequences of the ITS region and 48 of the *trn*L-*trn*F marker were applied. The topology of trees was obtained based on the Bayesian inference. *Schiedeella bajaverapacensis* was described and illustrated based on morphological evidence, and its taxonomic position was confirmed by phylogenetic analyses. The new entity is the 10th *Schiedeella* representative known from Guatemala.

## 1. Introduction

Guatemala is a relatively small country in terms of size, but its geographical and biological diversity is enormous. It is estimated that about 10,800 species of vascular plants can be found there [1], and many of them are endemic. The biodiversity richness of the Orchidaceae family in Guatemala is an attribute that few countries have, but for many years, it has been poorly studied. It ranges from a little more than 100 species mentioned by Bateman [2], 527 species mentioned by Ames and Correll [3], and 800 [4] as well as another 1237 species proposed by Archila [5]. By far, the most comprehensive work on Guatemalan orchid flora [1] has reported the presence of 223 genera and over 1200 species in this megadiverse country.

**Figure 1:**
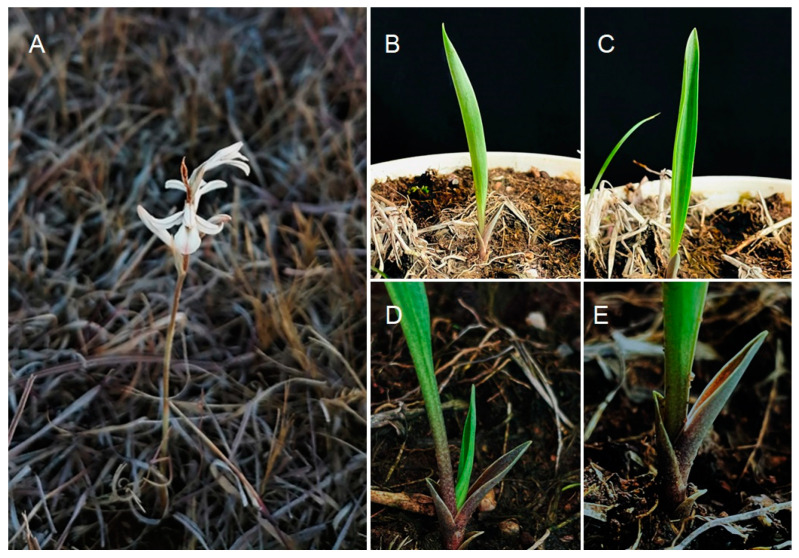
*Schiedeella bajaverapacensis*—living plant: (**A**) in situ, in the flowering stage; (**B**–**E**) ex situ, in the leafy stage. Photo. O. Alvarado (**A**), F. Archila (**B**–**E**).

The taxonomic position of the representatives of the tribe Spirantheae Lindl. (Orchidaceae) has been studied by many taxonomists during the past 200 years. However, different concepts of genera and species, as well as divergent interpretations of original diagnoses, have resulted in significant differences between the approaches of the authors. Genus *Schiedeella* Schltr. is one of the most controversial representatives of Spiranthinae. It is represented in Guatemala by nine species. While studying the orchid diversity in the department of Baja Verapaz, we found plants that clearly belonged to this taxon (Figure 1), but whose features did not fit any previously known species (Table 1). Hereby, we describe it as *Schiedeella bajaverapacensis* sp nov.

## 2. The History of Classification of *Schiedeella* Complex

The first attempt to systematize the subtribe Spiranthinae Lindl. was published in 1920 by Schlechter [6]. In his classification, Schlechter adopted uniform criteria for defining genera, assuming the most important to be the structure of the gynostemium and perianth morphology. He recognized 24 genera, of which 16 were newly segregated; among others, these were: *Deiregyne* Schltr. and *Schiedeella*. Unfortunately, Schlechter did not indicate the type of species for many of his new genera, which has later become the main reason for the differences in the taxonomic concepts between scientists working on this group. This classification has not been widely accepted by the scientific community, and the majority of the species in this subtribe described in the 1930s and 1940s were included in *Spiranthes sensu latissimo* [7].

The next attempt to clarify delimitations within the subtribe was proposed by Burns-Balogh [8]. According to her, Spiranthinae consists of approximately 250 species in 14 genera: *Brachystele* Schltr., *Buchtienia* Schltr., *Cyclopogon* C. Presl., *Deiregyne*, *Discyphus* Schltr., *Eurystyles* Wawra, *Hapalorchis* Schltr., *Odontorrhynchus* M.N. Correa, *Pelexia* Poit. *ex* Lindl., *Sarcoglottis* C. Presl., *Sauroglossum* Lindl., *Schiedeella*, *Spiranthes* Rich., and *Stenorrhynchos* Rich. *ex* Spreng. Using the rostellum/viscidium attachment as a distinguishing character, she grouped eleven of the above genera into four generic alliances. In this concept, the representatives of the *Brachystele* alliance have extremely reduced rostellum that may be completely fused to the oval viscidium. This assemblage includes *Brachystele*, *Sauroglossum*, and *Odontorrhynchus*. The *Pelexia* alliance is characterized by an elongate, broad, laminar rostellum that is adherent to the disc-like viscidium and embraces *Cyclopogon*, *Pelexia*, and *Sarcoglottis*. The *Spiranthes alliance*, which covers *Discyphus*, *Hapalorchis*, and *Spiranthes*, has a broad laminar rostellum that is completely fused to the fusiform viscidium. The *Stenorrhynchos* alliance consists of *Schiedeella* and *Stenorrhynchos*, which have subulate rostellum with an ensheathing viscidium. Almost at the same time, Garay [9] published his revision of the subtribe Spiranthinae. He distinguished 44 genera, 14 of which were new.

In 1995, Szlachetko [10] proposed to divide Spirantheae into six subtribes. In his classification, Spiranthinae counted 27 genera and embraced some taxa previously segregated by Burns-Balogh, such as *Brachystele*, *Buchtienia*, *Deiregyne*, *Eurystyles*, *Hapalorchis*, *Odontorrhynchus*, *Sauroglossum*, and *Spiranthes*. Genera such as *Schiedeella* and *Stenorrhynchos* (thus, all members of Burns-Balogh’s *Stenorrhynchos* alliance) were placed together with 19 other taxa in the newly established subtribe Stenorrhynchidinae Szlach.

*Schiedeella* Schltr. is one of the richest in species and at the same time is one of the most controversial genera within Stenorrhynchidinae (*sensu* Szlachetko [10]). Schlechter [6] considered *Schiedeella* to be closely related to *Cyclopogon*, however, differing from it in the morphology of the lip. The flowers of the two genera are fairly similar, but when studied in detail, distinct differences become evident, particularly in the structure of the rostellum and viscidium. As noted by Szlachetko [7], a distinct variance with Schlechter’s description is the structure of the rostellum and viscidium of *Schiedeella pyramidalis* Lindl. The remaining species, following the structure of the rostellum, perianth, and bracts, were divided into two distinct groups.

Regarding two species of eight, i.e., *S. pyramidalis* and *S. cobanensis* Schltr., proposed by Schlechter [6], Burns-Balogh [8,11] excluded them from *Schiedeella* and added eighteen further species, most of which were described after Schlechter’s death in 1925. She also designated *Spiranthes saltensis* Ames (=*S. durangensis* Ames & Schweinf.) as the type species for the genus. In 1986, Burns-Balogh [11] published the infrageneric classification of *Schiedeella*, distinguishing the following sections: *Parasitica* Burns-Bal., *Eriophora* Burns-Bal., and *Michoacana* Burns-Bal. The basis for this division was the type of floral bracts, the color of flowers and nectaries, and the morphology of the lip.

Garay [9] incorporated into *Schiedeella* only species that have a narrow-based rostellum and lip set on a flat claw with auricles at the base of the hypochile. As a result, he left only two taxa of the original set of species in the genus, adding four others. He also transferred the remaining species from *Schiedeella sensu* Burns-Balogh [8,11] to such genera as *Dithyridanthus* Garay, *Funkiella* Schltr., *Gularia* Garay, *Microthelys* Garay, *Physogyne* Garay, *Stenorrhynchos* and the majority to *Deiregyne*.

Szlachetko [7] pointed out that *Schiedeella sensu* Garay did not include *Spiranthes trilineata* Lindl., which from the point of view of the structure of the gynostemium and the perianth, quite markedly suggests *Schiedeella nagelii* (L.O. Williams) Garay. Instead, Garay created a new genus, *Gularia* Garay, for *Spiranthes trilineata* and *S. trilineata* var. *crenulata* (L.O. Williams) Szlach. and excluded the group *S. eriophora*/*S. velata* from *Schiedeella*. He treated them as representatives of *Deiregyne*. In Garay’s concept, *Gularia* differs from *Schiedeella* in the presence of a long, decurrent column foot, a pliable rostellum, and a claw fused with lateral sepals. However, according to Szlachetko [7], the column foot of *S. trilineata* is not longer than in other species of this genus. Studies of herbarium specimens have shown that the rostellum of this species is similar in texture to that of, for example, *S. llaveana* Schltr., and the fusion of the claw with the lateral sepals is present in various species of *Schiedeella*. In view of the lack of essential morphological conditions distinguishing the genus *Gularia*, Szlachetko [7] proposed to include it in *Schiedeella*.

Similarly to Burns-Balogh, Garay also noted that Schlechter had made certain suggestions as to the type of the genus *Schiedeella* and proposed *S. transversalis* A. Rich. & Galeotti as the generitype. Agreeing with Garay [9], Burns-Balogh [11] stated that different lectotypification of the genus would not alter her concept [7]. She treated *S. transversalis* as one of the species of *Schiedeella*.

In the present form, *Schiedeella* counts about 20 species whose distribution range covers practically the entire Central America and Antilles. The representatives of the genus are predominantly terrestrial plants, and their flowers are characterized by nearly erect gynostemium and a needle-like rostellum. The rostellum remnant is narrowly triangular and usually distinctly 3-dentate with the central tooth being the longest. The viscidium is nearly sheath-like, and the column foot is reduced. The lip is basally nearly erect or only slightly arcuate with a flat claw and more or less thickened auricles. Floral and cauline bracts are herbaceous or occasionally subscarious. Within the genus, based on the rostellum structure, three subgenera are being recognized: *Schiedeella*, *Schiedeellopsis* Szlach., and *Gularia* (Garay) Szlach. [12]. Subgenus *Schiedeella* counts 11 species and is characterized by long rostellum, apical vicidium, anther that is shorter than gynostemium and with an apex that reaches the viscidium’s upper part, and sepals that are free almost to the base. The subgenus *Schiedeellopsis* embraces four species, and based on lip shape, it is divided into two sections: *Schiedeellopsis* Szlach. (with three species) and *Gemmorchis* Szlach. (with a single species). This subgenus is characterized by a short rostellum, small, basal, oval, or ovate viscidium, an anther almost as long as gynostemium, and a rostellum remnant that is shortly three-dentate or subulate. The last subgenus, *Gularia*, covers three species and is distinguished by a long rostellum, anther apex that rarely reaches the base of the rostellum, and sepals whose basal part is connate, forming a short tube.

In 2016, Salazar et al. [13] segregated from *Schiedeella* the genus *Greenwoodiella* Salazar, Hernández-López & J. Sharma. The authors pointed out several morphological attributes, which in their opinion, distinguish the new genus from *Schiedeella*. These were: shoots connected by a slender rhizome, ovate or broadly elliptic, fleshy-coriaceous leaf with a rounded base, glossy dark green upper surface, and grayish-green underside with a purplish central vein. In terms of the floral features, the differences covered the proportionately shorter labellum, not surpassing the length of the lateral sepals. In the newly created taxon, they included *Greenwoodiella deserticola* Salazar, Hernández-López & J. Sharma, *G. micrantha* (Lex.) Salazar & R. Jiménez, *G. micrantha* (Lex.) Salazar et R. Jiménez var. *garayana* (R.González.) Salazar & R. Jiménez, and *G. wercklei* (Schltr.) Salazar & R. Jiménez.

## 3. Results

**Figure 2:**
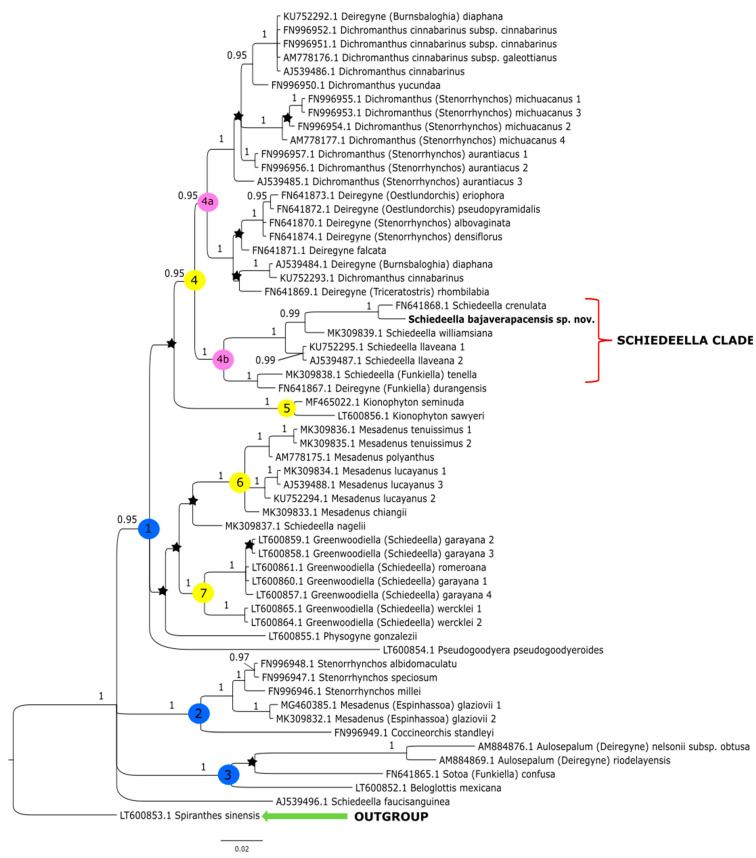
The phylogenetic tree presenting the position of *Schiedeella bajaverapacensis* sp. nov., obtained for the ITS1-5.8S-ITS2 region based on BI analysis. Posterior probability (PP) values are indicated above the branches and PP < 0.95 as an asterisk. While the numbers marked in circles on the nodes mean discussed clades.

**Figure 3:**
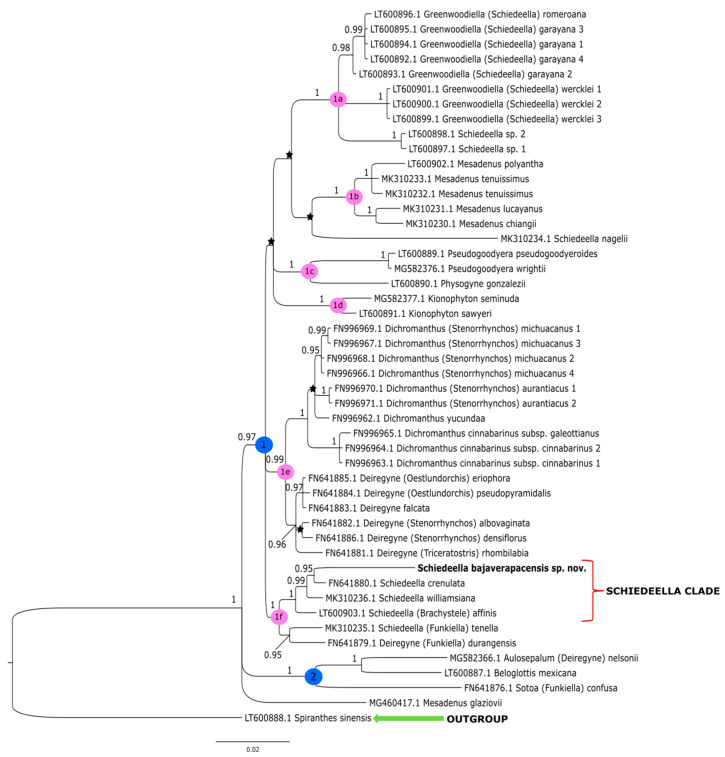
The phylogenetic tree presenting the position of *Schiedeella bajaverapacensis* sp. nov., obtained for the *trn*L-*trn*F region based on BI analysis. Posterior probability (PP) values are indicated above the branches and PP < 0.95 as an asterisk. While the numbers marked in circles on the nodes mean discussed clades.

The topologies obtained under these studies based on the Bayesian inference gave similar results to that of the maximum-likelihood method conducted by Salazar et al. [14]. Thus, we decided not to perform additional analyses based on other methods. The clades that have the strong support of bootstrap values in the trees of Salazar et al. [14] also have high posterior probability values in trees obtained by us from the Bayesian inference. In this article, we present the trees from the single data matrix (Figure 2 and Figure 3). Additionally, in Table 2, we list the statistical data on the particular matrices.

### 3.1. ITS Matrix

At the base of the tree obtained for the ITS dataset (Figure 2) *Spiranthes sinensis* (Pers.) Ames was placed as a representative of the outgroup. The remaining taxa are included together in one group within which there are three major clades (**1**–**3**). The sample is indicated as AJ539496.1, representing that *Schiedeella fauci-sanguinea* (Dod) Dod was not grouped with other species of this genera, only placed as a polytomy branch within a major clade of the ingroup. The first, clade **1** with high posterior probability support (PP = 0.95), embraces most of the analyzed taxa and includes four smaller groupings (clades **4**–**7**). Clade **4** (PP = 0.95) is split into two subclades: **4a** (PP = 0.95) comprising representatives of *Dichromanthus* Garay and *Deiregyne* Schltr., and a sister subclade **4b** (PP = 1), which includes species of *Schiedeella*. In subclade **4b** is placed the new species—*Schiedeella bajaverapacensis*. It forms a consistent and strongly supported (PP = 1) group with *S*. *crenulata* (L.O.Williams) Espejo & López-Ferr., *S*. *williamsiana* Szlach. & al., and *S*. *llaveana* Lindl. The latter species are sampled twice. *Deiregyne durangensis* (Ames & C.Schweinf.) Garay (FN641867.1) was also ranked in this subclade. Clade **5** (PP= 1; Figure 2) embraces only two species representing *Kionophyton* Garay while clade **6** (PP = 1) includes members of the genus *Mesadenus* Schltr. The last group within the major clade **1** (PP = 0.95) is represented by the members of *Greenwoodiella* Salazar, Hern.-López & J.Sharma (subclade 7, PP = 1).

In clade **2** (PP = 1, Figure 2), *Coccineorchis standleyi* (Ames) Garay is a sister taxon to the representatives of *Mesadenus glaziovii* (Cogn.) Schltr. (=*Espinhassoa glaziovii* (Cogn.) Salazar & J.A.N. Bat.), which was sampled twice, and species of *Stenorrhynchos* Rich. *ex* Spreng. The last of the major ones, clade **3** (PP = 1, Figure 2), embraces *Beloglottis mexicana* Garay & Hamer and *Sotoa confusa* Salazar, both as polytomic branches, and a small group that includes two species of *Aulosepalum* Garay.

### 3.2. Plastid Matrix

The tree obtained for the plastid region (Figure 3) showed slightly different results. In the ingroup, we can distinguish two major clades, marked as **1** and **2**. A sample of *Mesadenus glaziovii* indicates that MG460417.1 was not included with one group of other representatives of this genera but constitutes the polytomic branch. Clade **2** (PP = 1) embraces *Sotoa confusa* as a sister taxon to the group that includes *Aulosepalum nelsonii* (Greenm.) Garay and *Beloglottis mexicana*, which corresponds to clade **3** on the tree, performed for the ITS marker (Figure 2). However, the grouping of analyzed taxa at the level of smaller subclades is much more similar to the results obtained for the ITS matrix. Clade **1** (PP = 1) is divided into six strongly supported groups (clades **1a**–**1f**). The representatives of *Greenwoodiella* form subclade **1a** (PP = 1), and it almost corresponds to clade **7** (PP = 1) on the ITS tree (Figure 2). Subclade 1a differs in the presence of two samples of *Schiedeella* sp. A similar situation is observed for subclade **1b** (PP = 1) and **1d** (PP = 1) that respectively embrace the species of *Mesadenus* and *Kionophyton*. In this case, *Pseudogoodyera* is sampled by two species, and this probably caused them to form a strongly supported subclade **1c** (PP = 1) together with *Physogyne gonzalezii* while on the ITS tree; these taxa form polytomous branches. The taxa of *Dichromanthus* and *Deiregyne* are grouped together (subclade **1e**, PP = 0.99) similarly on the ITS tree (Figure 2). However, subclade **1e** (Figure 3) separates into two strongly and monophyletic groups, and one of these includes species of *Deiregyne* (PP = 0.96) and *Dichromanthus* (PP = 1). In the last subclade **1f** (PP = 1), the new species *Schiedeella bajaverapacensis* is placed with one group of other representatives of *Schiedeella*. As in the ITS tree (Figure 2), *S*. *bajaverapacensis* is closely related to *S*. *crenulata* (PP = 0.95). The results for both nuclear and plastid datasets showed that the phylogenetic position of the new taxon is within the *Schideella* group. However, the *trn*L-*trn*F tree (Figure 3) in subclade **1f**, the *Schiedeella* group, ranked *S. affinis* (*Brachystele*), with strong probability support (PP = 1). Unfortunately for the ITS marker, we did not have a sample for this species, and thus, we cannot compare this result.

### 3.3. Taxonomic Treatment

**Figure 4:**
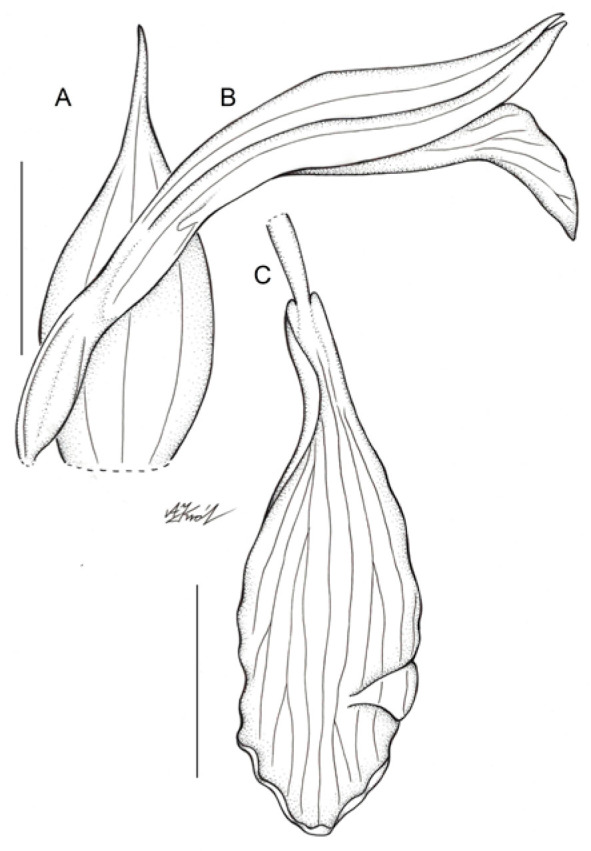
Floral parts of Schiedeella bajaverapacensis: (**A**) floral bract; (**B**) flower—side view; (**C**) Lip. Scale bars—5 mm. Drawn from the holotype by A. Król.

**Figure 5:**
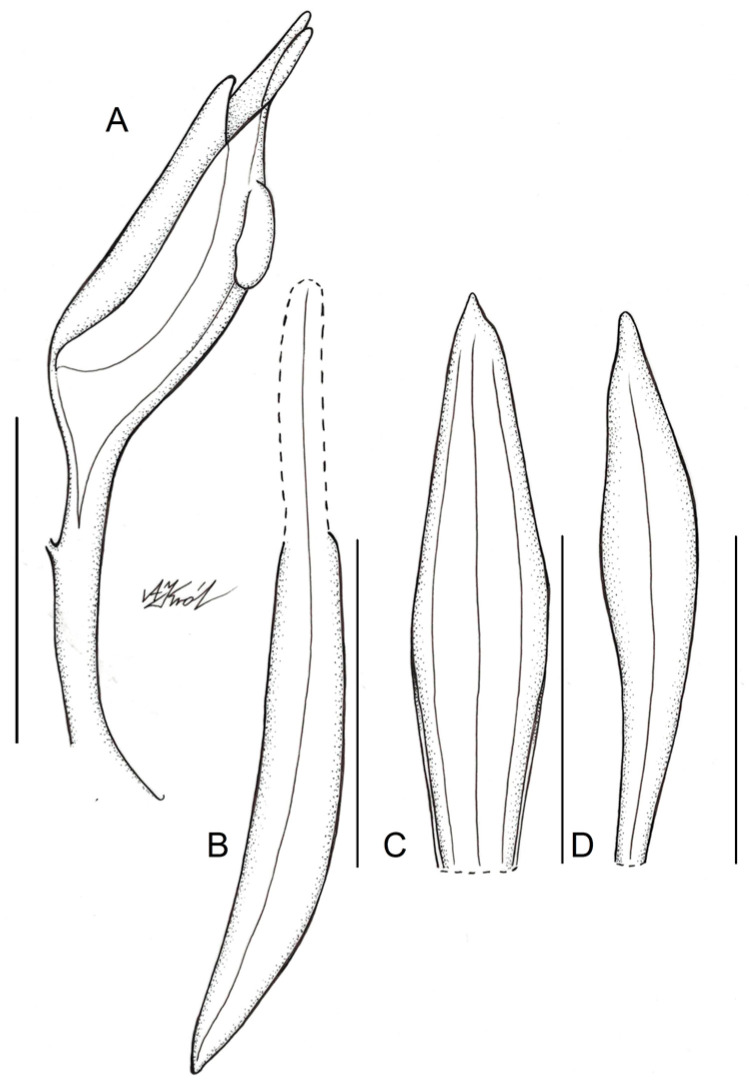
Floral parts of Schiedeella bajaverapacensis: (**A**) gynostemium—side view; (**B**) lateral sepal; (**C**) dorsal sepal; (**D**) petal. Scale bars—5 mm. Drawn from the holotype by A. Król.

***Schiedeella bajaverapacensis*** Archila & Szlach., sp. nov. (Figure 4 and Figure 5).

TYPE: GUATEMALA. Departamento de Baja Verapaz. Municipio de Salamá, a 950 m asl. Creciendo junto a *Melocactus curvispinus*, *Opuntia cochenillifera* y *Cohniella brachyphylla*. Colectada en enero 2022 por Otto Alvarado (Recorded by Archila & Szlachetko) (HOLOTYPE: BIGU!, ISOTYPES: BIGU!, UGDA-DLSz!, UGDA-DLSz!—liquid).

The new species appears to be similar to *Schiedeella crenulata* and *S. trilineata* var. *undulata*. *S. bajaverapacensis* differs from both mentioned taxa in the lip form, which is elliptic-ovate in the general outline, with a narrow basal part, shortly and obscurely auriculate, and an apical third of lamina somewhat undulate along the margins. The lip of *S. crenulata* is indistinctly divided into hypochile and epichile, which is ligulate with crenulate margins. The lip of *S. trilineata* var. *undulata* is oblanceolate in outline, is obtuse, and is widest in 2/3 of its length with distinctly undulate margins in the apical half.

Description: Plants terrestrial, small. Stem ca. 10 cm tall, erect, delicate, completely glabrous, enveloped in three sheaths, leafless at anthesis. Sheaths ovate-lanceolate, acuminate, glabrous, semi-transparent. Leaf single, at the beginning protected with three bracts, until it emerges from the ground; blade ca. 6.2 cm long and 0.8 cm wide, elliptic, microscopically obliquely acute. Protective bracts with sharp apex, gray in the abaxial part and brown in the adaxial part, of different sizes: 0.5 cm long and 0.3 cm wide the external one, 0.9 cm long and 0.5 cm wide the intermediate one and 1.7 cm long and 0.4 cm wide the internal one. Inflorescence ca. 2.5 cm long, laxly five-flowered. Flowers white, sepals and petals with prominent, grayish central vein, lip pure white with pinkish suffusion in the center and grayish central vein. Floral bracts 10 mm long, obliquely ovate, long-acuminate, with three prominent veins, glabrous, semi-transparent, whitish. Ovary 4–5 mm long, glabrous, white. Sepals connate forming a prominent tube in the basal quarter, somewhat swollen. Dorsal sepal free part 9 mm long, 2 mm wide, lanceolate, acute, basally connate with the gynostemium, somewhat concave in the center, three-veined, glabrous. Petals 8.5 mm long, 1.5 mm wide, linear-oblanceolate, acute, subfalcate, adnate to the dorsal sepal forming a kind of galea, one-veined. Lateral sepals free part 8 mm long, 1.5 mm wide, linear, acute, subfalcate, one-veined, glabrous. Lip clawed; claw 3 mm long, narrow, adnate to the sepaline tube; blade 11 mm long in total, 3.2 mm wide when spread, elliptic-ovate in general outline, basally shortly auriculate, auricles somewhat thickened, apical third of lamina somewhat undulate along margins, apex obtuse, multiveined, thin, glabrous. Gynostemium 11.5 mm long, erect, somewhat swollen in the apical half. Column foot 1.5 mm long, oblique. Anther 4.5 mm long, oblong-ovate. Rostellum 2.1 mm long, erect, subulate. Viscidium 1.1 mm long, sheath-like. Clinandrium spacious.

Etymology: In reference to the Department of Baja Verapaz, where new species was collected.

Ecology and distribution: Known only from the type locality in the department of Baja Verapaz, Guatemala. It grew in an open area at an altitude of 950 m asl, together with *Melocactus curvispinus* Pfeiff., *Opuntia cochenillifera* (L.) Mill., and *Cohniella brachyphylla* (Lindl.) Cetzal & Carnevali. Flowers in January.

Conservation status: According to the IUCN red list criteria [15], *S. bajaverapacensis* was assessed as data deficient (DD).

## 4. Discussion

**Figure 6:**
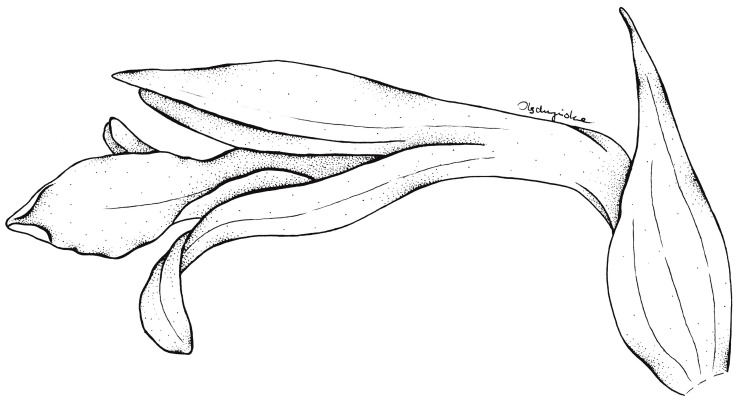
Flower of Schiedeella crenulata—drawn from the holotype (Forderstrom 2592, deposited at AMES). Drawn by N. Olędrzyńska.

**Figure 7:**
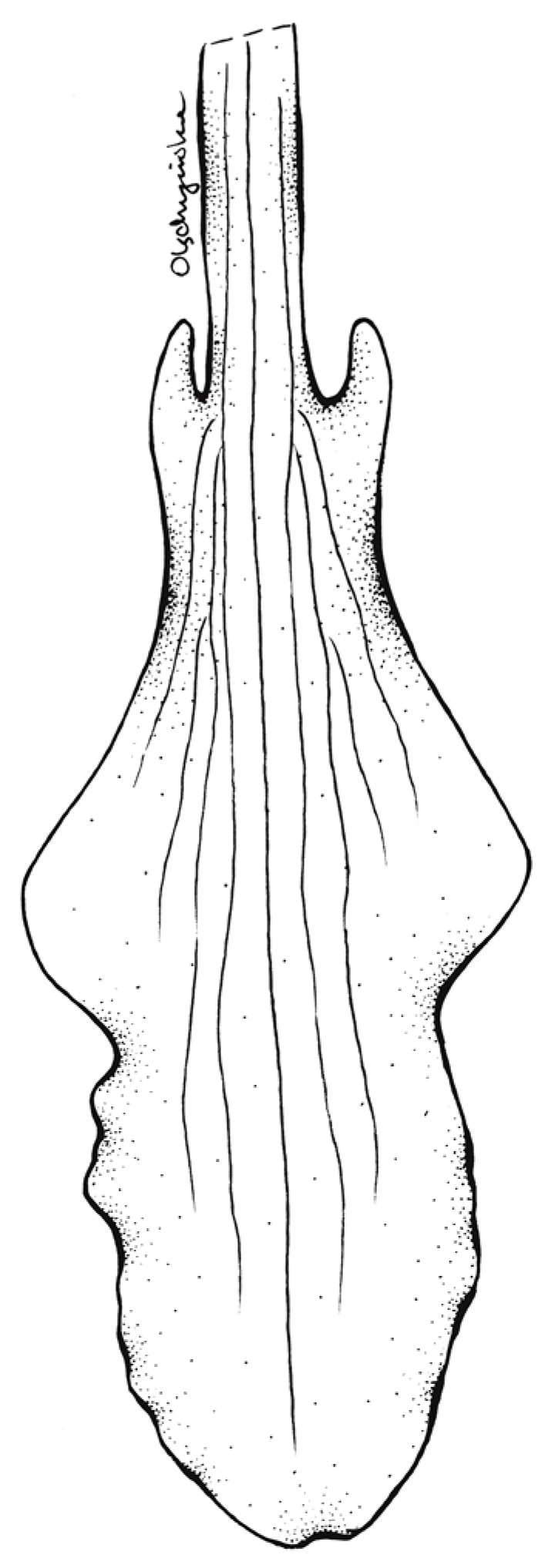
Lip of Schiedeella crenulata—drawn from the holotype (Forderstrom 2592, deposited at AMES). Drawn by N. Olędrzyńska.

According to the results of the molecular analyses, *Schiedeella bajaverapacensis* appears to be genetically related to *S. crenulata* (L.O.Williams) Espejo & López-Ferrari from the southern states of Mexico (Chiapas, San Luis Potosi, Puebla) and Honduras. On the two trees we obtained (Figure 2 and Figure 3), these species are grouped into a single clade with strong posterior probability support. Both species share similar habits, as all taxa of the subgenus *Gularia* (Garay) Szlach., i.e., short, few-flowered inflorescences, leafless at anthesis (Table 3. We had the opportunity to study the type material of this taxon—*Fröderström 2592*—stored at AMES (Figure 6 and Figure 7). The lip appears to be somewhat different from the one depicted by Williams on the herbarium sheet. Unlike our new entity, the lip of *S. crenulata* (Figure 7) is indistinctly divided into hypochile and epichile; the hypochile is deltoid in outline and the narrower epichile is ligulate, with an obtuse apex and crenulate margins. Unfortunately, we do not have access to any material of *S. trilineata* var. *undulata* Szlach., which is morphologically quite similar, to the molecular study. This variety has been described based on material collected in Guatemala, Dept. Guatemala, “Vara de San Jose”. In both taxa, we can observe an undivided lip. In the var. *undulata*, the lip is oblanceolate in outline, obtuse, and widest in 2/3 of its length with distinctly undulate margins in the apical half. Basal auricles are prominent and thick, and the width of the lip at this point is similar to the widest part of the lip. The lip of *S. bajaverapacensis* is elliptic-ovate in the general outline, the basal part is narrow and shortly and obscurely auriculate, and the apical third of lamina is somewhat undulate along the margins.

## 5. Materials and Methods

**Figure 8:**
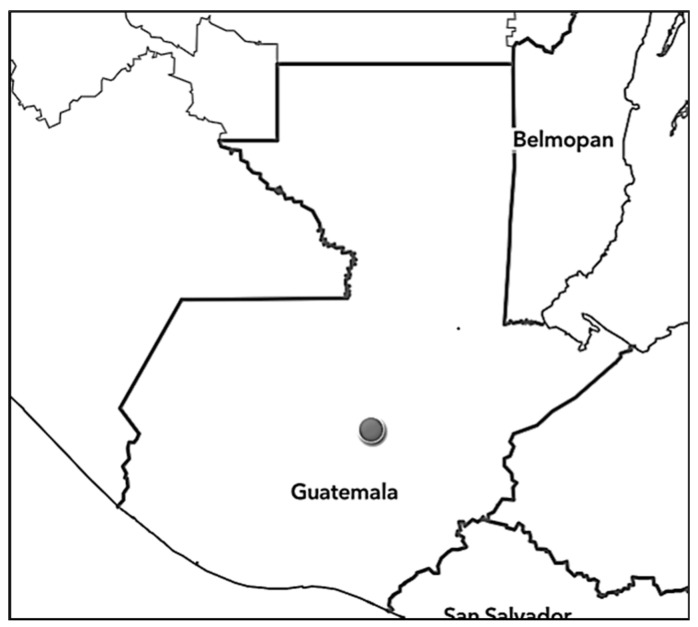
The approximate location where *Schiedeella bajaverapacensis* was found.

Plants were collected in the Salamá municipality, Department of Baja Verapaz (Central Guatemala; Figure 8) in January 2022 by Otto Alvarado. After collection, herbarium specimens were prepared and deposited in the herbarium BIGU and UGDA.

### 5.1. Morphological Study

Specimens were investigated according to standard procedures. The first step consisted of creating the database of the information from herbarium labels and taking digital photographs of the sheet. Vegetative parts, i.e., rhizomes, roots, pseudobulbs, leaves, shoots, inflorescences, and floral sheaths, were accurately measured and described. The flower was dissected from the inflorescence and after rehydration was subjected to detailed research using a stereoscopic microscope. In case of doubts regarding a given feature, a larger number of flowers was tested, if possible. Each part of the perianth was accurately measured, drawn, and described. Particular attention was paid to the examination of the lip and the column. The morphology of these structures is extremely important: in the case of the lip, its shape, nervousness, size, the presence of various kinds of swellings, folds, and narrowings. When examining the morphology of the column, particular attention was paid to the construction of viscidium, rostellum, clinandrium, and proportions of the length of the column’s foot to the column, possibly its adhesion with the lateral outer petals of the perianth.

The collected documentation was compared with the type specimens, diagnoses, and, if available, original drawings. Most of the materials were collected and examined during visits to European and American herbaria: AMES, B, BIGU, C, CAY, COL, HJBG, HUA, K, MA, MO, NY, P, RPSC, QCA, QCNE, US, VEN, W, WU (acronyms adopted from Index Herbariorum [16]). We carefully analyzed the data presented in the available literature, especially floras published by Bateman [2], Ames and Correll [3], Archila [4] and Archila et al. [1].

### 5.2. Molecular Analysis

GenBank accession numbers for the sequences of the new taxon are listed in Table 4. A list of the taxa with their accession numbers is included in Appendix A In order to determine the phylogenetic relationships of the new species, taxa grouped together with *Schiedeella* based on the results previously published by other researchers were used [13,14].

**DNA Isolation.** Total genomic DNA was extracted using the DNA Sherlock AX Kit (A&A Biotechnology, Gdansk, Poland) following the manufacturer’s protocol. First, the sample was dried in silica gel [17]. For isolation, 28 mg of the dried material (fragment of the flower) was taken. The pellet of DNA was suspended in 50 µL of TE buffer. The quantity and purity of the isolated DNA were determined and checked using NanoDrop One of Thermo Scientific, Waltham, MA, USA.

**PCR and sequencing reactions.** The amplification was performed using StartWarm HS-PCR Mix (A&A Biotechnology, Poland) following the manufacturer’s protocol. The total volume of the sample was 25 µL, containing 1 µL template DNA (∼10–100 ng) and 0.5 µL of 10 µM of each primer. Parameters for the PCR reaction for nrITS (ITS1 + 5.8S + ITS2) and for the plastid region *trn*L-*trn*F were the same as Martin et al. [18]. Tested products of the amplification were purified by applying the Clean-Up Concentrator Kit (A&A Biotechnology, Poland). The final elution was performed in 30 µL of nuclease-free water. Then, purified PCR products were sequenced by Macrogen (Seoul, Republic of Korea-http://dna.macrogen.com/eng/). For the amplification and sequencing, we utilized the same pairs of primers: for the ITS region, 101F and 102R [19], while for the plastid marker *trn*L-F using primers *trn*L-c and *trn*L-f [20].

**Phylogenetic reconstruction.** For phylogenetic reconstruction, we applied 59 sequences of the ITS region and 48 of the *trn*L-*trn*F marker, representing 14 genera and 43 species of Spiranthinae (including new species) of which 12 occur in Guatemala. Some species were sampled more than once. In selecting the taxa of ingroup and outgroup for our analysis, we relied on the results obtained by Salazar et al. [14]. First, we aligned all sequences for single markers using the “align” option according to the MUSCLE algorithm [21] with SeaView [22]. The substitution model for each examined region was calculated with MrModeltest 2.2 [23]. For both datasets, the same best fit model (GRT + G + I) applying AIC (Akaike information criterion) was selected. The Bayesian inference (BI) was performed using four Markov-chain Monte Carlo (MCMC) chains and four independent runs with MrBayes v. 3.2.7a [24]. Each run started from different random trees to ensure that individual runs converged to the same result. Three million generations for both matrices per run were used with sampling 1 tree for every 100 generations until the average standard deviation of the split was smaller than 0.01. Then, the initial 25% of the sampled generations of each chain was as burn-in. Saved trees were summarized in a 50% majority-rule consensus tree and were edited with FigTree v.1.4.4 (http://tree.bio.ed.ac.uk/software/figtree/) and Inkscape (https://inkscape.org/release/inkscape-1.0.2/). The node’s confidence for the obtained trees was assessed by posterior probabilities (PP), which were considered strongly supported when equal to or higher than 0.95 [25].

## Figures and Tables

**Table 1:** Comparison between the morphological features of Guatemalan representatives of the genus *Schiedeella*.

	*S. esquintlensis*	*S. jeanmulleri*	*S. llaveana*	*S. parasitica*	*S. romeroana*	*S. schlechteriana*	*S. trilineata*	*S. trilineata var. thelymitra*	*S. valerioi*
Name’s authors	Szlach., Rutk. and Mytnik	Szlach., Rutk. and Mytnik	(Lindl *ex*. Benth) Schltr.	(A. Rich. and Galeotti) Schltr.	Szlach.	Szlach. and Shevia	(Lindl.) Burns-Bal.	(Rchb. f.) Szlach.	(Ames and C. Schweinf.) Szlach. and Sheviak
Distribution	Esquintla	Esquintla	Baja Verapaz, San Marcos, El Progresso	Chimaltenango, San Marcos	Huehuetenango	Totonicapán	Chimaltenango, Chiquimula, Guatemala, Huehuetenango, Zacapa	Chiquimula	Chimaltenango, Quetzaltenango
Stem	10 cm high, delicate, erect, glabrous	Ca. 20 cm high, very delicate, erect, glabrous, 6–7 sheaths	19–16 (66) cm high, usually delicate, erect, glabrous, 5–7 sheaths	10–25 (38) cm high, delicate, erect, usually glabrous, rarely sparsely glandular along spike, 5–7 sheaths	35 cm high, delicate, erect, tinged reddish, sparsely glandular under and within the spike, 9 sheaths	6.5–8 cm tall, erect, glabrous below, glandular above the uppermost bracts	6–20.5 cm high, erect, delicate, glabrous, whitish or brownish, 4–8 sheaths		15–43 cm high, rather delicate, glabrous, only in the upper part glandular, 5–7 sheaths
Sheaths	Imbricate, semiscarious, semitransparent cauline	Cauline, herbaceous, thin, delicate, the lower ones imbricate, the upper ones shorter than internodes, glabrous	Cauline, more or less adnate to the stem, acute, herbaceous, the lower ones longer than internodes, the others shorter	Cauline, adnate to the stem, acuminate, herbaceous, sometimes with hyaline margins, shorter than internodes, except the lowermost, glabrous	Cauline, tubular, acuminate, tightly adnate to the stem, herbaceous, with hyaline margins, glabrous	Cauline, longer than the internodes (the uppermost reaching the inflorescence), sharply pointed, herbaceous, with parallel veins	Cauline, more or less adnate to the stem, sharply pointed, herbaceous with hyaline margins, green, pink or brownish		Cauline, herbaceous, very thin, acute, more or less adnate to the stem
Leaves	Unknown	Basal, 2–3, petiole to 10 cm long, narrow; blade to 8 cm long and1 cm wide, narrowly lanceolate, acute	Basal, petiolate; petiole up to 9 cm long, narrow; blade ca 5 cm long and 1 cm wide, lanceolate, broadly lanceolate to oblong-elliptic, acute	Basal, petiolate; petiole 4.5 cm long, narrow; blade up to 6 cm long, probably lanceolate, acute	Single, basal, petiolate; petiole about 4 cm long, narrow; blade 5 cm long, 2.5 cm wide, ovate-lanceolate, acute, attenuate towards the base	Basal, fugaceous, otherwise unknown	Basal, petiolate; petiole 1.5–2 cm long		Basal, 1–2, petiolate; petiole 0.8–4 cm long, narrow; blade 1–4 cm long and 0.7–1 cm wide, ovate-lanceolate, acute
Inflorescence	4–5 cm long, laxly 6–8-flowered	3–4 cm long, laxly 6–8-flowered	5–23 cm long, 8–19-flowered, lax, secund	2–10 (13) cm long, (3) 4–10 (16) flowered, rather lax to relatively dense, subsecund	9 cm long, ca 20-flowered, lax	1.5–2 cm long, laxly 2- or 3-flowered, the axis glandular	1–7 cm long, (1) 2–16-flowered, dense, sometimes capitate, to lax		2–10 (13) cm long, 3–9-flowered, lax
Flowers	Middle-sized, lip white, sepals dull pinkish-white with prominent brownish nerves	Rather small, lip white with dull yellowish throat, sepals dull pinkish or brownish with prominent nerves	Small, tubular, perianth brownish-red or grey-green, lip white, sometimes with a green base	Small, tubular, with slightly divergent apices, perianth green, pink or reddish, lip white with 5 olive-brown nerves or greenish-yellow front	Horizontal, tiny, tubular	Small, tubular, white, perianth segments longer than ovary	Tubular, apical ones erect, tubular, divergent at the apex only, glabrous; usually white with green nerves on petals, rarely dull white or white with pink deposit, or yellow		Tubular, divergent at the apex, perianth dull, greenish-white to pale pink or pinkish red-brown
Floral bracts	Slightly longer than ovary, glabrous, herbaceous with hyaline margins, with 3 prominent nerves, dull pinkish-white	Distinctly longer than ovary, glabrous, herbaceous with hyaline margins, with 3 prominent nerves, dull pinkish or brown-ish	9–20 mm long, broadly lanceolate, acute, herbaceous	5–16 mm long, ovate-lanceolate, acuminate, herbaceous, glabrous	5–7 mm long, ovate, apex obliquely lanceolate, acute, herbaceous, glabrous	10 mm long, about twice as long as the ovary, thin, herbaceous, semitranslucent, acuminate, with 3 branched nerves	5–21 mm long, as long as or 1.5 times longer than ovary, ovate to broad lanceolate, acute, semi-transparent, thin, herbaceous with hyaline margins		7–22 mm long, about 1.5 times longer than ovary, ovate-lanceolate, acute, thin, semi-transparent, pale green, usually 3-nerved
Ovary	6 mm long, twisted, glabrous, dull pinkish-white	5 mm long, twisted, sparsely glandular, dull pinkish to brownish	5–10 mm long, cylindrical, glabrous, or sparsely glandular at the apex only	3–9 mm long, narrowly cylindrical, apically bent forwards, sparsely glandular at the apex	4–6 mm long, cylindrical, glabrous	5 mm long, sparsely glandular	4–7 mm long, slender, glabrous		4.2–11 mm long, cylindrical, narrow, glabrous or almost glabrous
Dorsal sepal	15 mm long,1.6 mm wide, linear with lanceolate, acute apex, 1-nerved	9 mm long, 1.4 mm wide, linear with lanceolate, acute apex, 1-nerved	(4.2) 7–10.4 mm long, 1–2 mm wide, lanceolate to linear-lanceolate, concave above the base, broadest near the middle, glabrous or sparsely glandular at the base, 1 or obscurely 3-nerved	5–8 mm long, 1–1.9 mm wide, linear-lanceolate, concave above the base, 1-nerved	5 mm long, 1 mm wide, oblong-lanceolate, obtuse, thin, semi-transparent, 1-nerved	6 mm long, 1.5 mm wide, lanceolate, acute, glabrous, indistinctly 3-nerved	9.5–14 mm long, 1.6–3.2 mm wide, usually lanceolate to oblong-lanceolate, broadest in the middle or beneath, acute, near the apex slightly concave, 1- or rarely 3-nerved		4.3–7.2 mm long, 1.1–2 mm wide, usually broad- or oblong-lanceolate, acute, rarely obtuse, 1-nerved
Petals	11 mm long, 0.3–0.4 mm wide, almost filiform, acute,1-nerved, adnate to the dorsal sepal forming a prominent galea	7.2 mm long, 0.5 mm wide, linear, acute, 1-nerved, adnate to the dorsal one forming a prominent galea.	(4.1) 6.7–9 mm long, (0.5) 0.8–1.2 mm wide, linear to narrow-spathulate, widest in the upper part, rounded or obtuse at the apex, sometimes on the outer margin near the apex minutely dentate	4.5–7.3 mm long, 0.5–1 mm wide, linear-oblanceolate, widest in the upper part, subacute, 1-nerved	6 mm long, 1.3 mm wide, oblong, subobtuse, slightly asymmetric, 1-nerved	5 mm long, 1.2 mm wide, indistinctly falcate-banded, very thin, relatively broad, the apical margin erose, 3-nerved	9.5–14 mm long, 0.8–1.2 mm wide, usually linear to linear-lanceolate, sigmoid or falcate, acute or obtuse, 1-nerved		4–6.1 mm long, 0.8–1.5 mm wide, linear-oblanceolate or linear- spathulate, slightly sigmoid or falcate, acute or obtuse, at the apex irregular minutely indented, 1-nerved
Lateral sepals	17 mm long, 2 mm wide, oblique at the base, linear with oblanceolate apical half, acute to sub-obtuse at apex,1-nerved	8 mm long, 1.2 mm wide, slightly oblique, linear with lanceolate, acute apex, 1-nerved	(3.8)7–9.5 mm long, (0.8)1–1.9 mm wide, linear-lanceolate, slightly falcate, acute, glabrous or sparsely glandular at the base, 1-nerved	4.6–9 mm long, 0.9–1.2 mm wide, linear-lanceolate, acute, falcate, 1-nerved	4.9 mm long, 0.6 mm wide, oblong-spathulate, obtuse, very thin, 1-nerved	5.2 mm long, 1.3 mm wide, narrowly triangular, acute, glabrous, 1-nerved	10–14.5 mm long, 1–2.9 mm wide, linear to linear-oblanceolate, acute or obtuse, broadest in the upper part, slightly sigmoid or falcate, 1-nerved		5–6.4 mm long, 0.8–1.6 mm wide, oblong to narrowly lanceolate, acute, erect or slightly sigmoid, 1-nerved
Lip	On prominent claw, constricted distinctly in the apical fifth; hypochile 13 mm long, 2.9 mm wide, widest below the apex, with prominent digitate auricles at the base, glandularin the center on the upper surface; isthmus shallow; epichile 2.8 mm long, 3 mm wide, almost rounded, sub-triangular at apex, with slightly undulate margins	On prominent claw, constricted distinctly in the apical third; hypochile 7 mm long, 3.2 mm wide, widest at the apex, with prominent digitate auricles at the base, glandular on the upper sur-face; isthmus acute; epichile 3.2 mm long, 4.1 mm wide, almost rounded to slightly transversely elliptic, rounded at the apex, with prominently crispate margins	On claw, divided into hypo- and epichile; in natural position canaliculate with epichile erect or bent down; hypochile 3.5–6 (7.5) mm long, 1.9–4 (4.5) mm wide, more or less rectangular in outline, usually as wide at the base as near the apex, with two prominent, thickened basal auricles; epichile 2–4 mm long, 2–4.1 mm wide, elliptic to ligulate, obtuse	Shortly clawed; lamina distinctly constricted above the middle—hypochile 3.5–7 mm long, 1.8–4 mm wide, shallowly constricted above the basal auricles, than oblong-obovate, widest above the middle, with rounded lateral margins; epichile 1.7–3.7 mm long, 1.9–3.4 mm wide, elliptic, rounded in the apex, irregularly denticulate	Shortly clawed; claw 0.6 mm long, narrow; lamina constricted near apical third—hypochile 4 mm long, 2.2 mm wide, rectangular slightly thickened in the middle, the basal auricles rounded; epichile 2 mm long, 1.9 mm wide, rounded, minutely dentate, papillate, subobtuse	Rather narrow, oblong, with distinctly dendritic proliferation of nerves, shortly clawed, constricted near the middle—hypochile 3 mm long, 2.1 mm wide, at the base the upper surface glandular with two fleshy, very small auricles bent toward the apex of the lip, hypochile gradually tapered to epichile, more or less rhombic, the side lobes triangular; epichile 2 mm long and wide, oval, obtuse, the margin very minutely but distinctly erose	Usually distinctly constricted below the middle, on long claw agglutinate to the lateral sepals—hypochile 5.5–12.5 mm long, 1.8–5.5 mm wide, greatly varied in shape -oblong-triangular, obovate lanceolate, ovate; broadest below apex at the base two, fleshy, pubescent auricles, in the center slightly thickened and pubescent, side lobes rounded, rarely triangular, sharply pointed agglutinate to the clinandrium; epichile 2–5 m long, 2–4.6 mm wide, reniform or cordate, obtuse or sometimes acute, broadest at the base, papillate usually irregularly indented, sometimes folded; if the lip undivided—usually oblanceolate, obtuse below the center with small lobes	With distinct constriction in the lower part dividing into hypo- and epichile. Epichile more or less folded, distinctly smaller than hypochile	Shortly clawed; claw narrow, linear; lamina indistinctly divided, in natural position canaliculate, erect with epichile strongly bent down- hypochile 2.9–4.5 mm long, 1.5–2.1 mm wide, 5-nerved, rectangular, at least 2 times longer than wide, truncate at the base, with slightly thickened auricles, in the center slightly thickened; epichile 1–2 mm long, 1–1.9 mm wide, 3-nerved, lingulate, ovate to transversely ovate, acute or obtuse, minutely irregular indented, papillate
Gynostemium	13 mm	7 mm	7–8 mm	3.5–5 mm	3.5 mm	2 mm	7–13.5 mm		4–5 mm
Viscidium	1 mm	0.5 mm	0.4 mm	0.4 mm	Not found	1 mm	0.8–1 mm		0.5 mm

**Table 2:** Statistical data for each single dataset.

Dataset	No. of Taxa	Total Characters	Constant Characters	Informative Characters
ITS1-5.8S-ITS2	59	703	466	183
*trn*L-*trn*F region	48	1582	1228	150

**Table 3:** Summary of the morphological differences between *Schiedeella bajaverapacensis* and genetically related *S. crenulata*.

	*S. bajaverapacensis* sp. nov.	*S. crenulata*
Stem	10 cm high, erect, delicate, completely glabrous, 3 sheaths	6–20.5 cm high, erect, delicate, glabrous, whitish or brownish, 4–8 sheaths
Sheaths	Cauline, ovate-lanceolate, acuminate, glabrous, semi-transparent	Cauline, more or less adnate to the stem, sharply pointed, herbaceous with hyaline margins, green, pink or brownish
Leaves	Ca. 6.2 cm long and 0.8 cm wide, elliptic, microscopically obliquely acute	Basal, petiolate; petiole 1.5–2 cm long, blade unknown
Inflorescence	Ca. 2.5 cm long, laxly 5-flowered	1–7 cm long, (1)2–16-flowered, dense, sometimes capitate, to lax
Flowers	More or less widely opened at the apex, white, sepals and petals with prominent, grayish central vein, lip pure white with pinkish suffusion in the center and grayish central vein	Tubular, apical ones erect, divergent at the apex only, glabrous; usually white with green nerves on petals, rarely dull white or white with pink deposit, or yellow
Floral bracts	10 mm long, obliquely ovate, long-acuminate, with 3 prominent veins, glabrous, semi-transparent, whitish	5–21 mm long, as long as or 1.5 times longer than ovary, ovate to broad lanceolate, acute, semi-transparent, thin, herbaceous with hyaline margins
Ovary	4–5 mm long, glabrous, white	4–7 mm long, slender, glabrous
Dorsal sepal	Free part 9 mm long, 2 mm wide, lanceolate, acute, basally connate with the gynostemium, somewhat concave in the center, 3-veined, glabrous	9.5–14 mm long, 1.6–3.2 mm wide, usually lanceolate to oblong-lanceolate, broadest in the middle or beneath, acute, near the apex slightly concave, 1- or rarely 3-nerved
Petals	8.5 mm long, 1.5 mm wide, linear-oblanceolate, acute, subfalcate, adnate to the dorsal sepal forming a kind of galea, 1-veined	9.5–14 mm long, 0.8–1.2 mm wide, usually linear to linear-lanceolate, sigmoid or falcate, acute or obtuse, 1-nerved
Lateral sepals	Free part 8 mm long, 1.5 mm wide, linear, acute, subfalcate, 1-veined, glabrous	10–14.5 mm long, 1–2.9 mm wide, linear to linear-oblanceolate, acute or obtuse, broadest in the upper part, slightly sigmoid or falcate, 1-nerved
Lip	Clawed; claw 3 mm long, narrow, adnate to the sepaline tube; blade 11 mm long in total, 3.2 mm wide when spread, elliptic-ovate in general outline, basally shortly auriculated, auricles somewhat thickened, apical third of lamina somewhat undulate along margins, apex obtuse, multiveined, thin, glabrous	Clawed; claw 3 mm long, narrow, adnate to the sepaline tube; blade 9 mm long in total, 4.2 mm wide when spread, multiveined; hypochile deltoid, 5 × 4.2 mm, basally auriculated; epichile 4 × 2.9 mm, logulate, obtuse, margins crenulate.
Gynostemium	11.5 mm	9 mm
Viscidium	1.1 mm long	0.9 mm

**Table 4:** The accession numbers of GenBank for the sample of *Schiedeella bajaverapacensis*.

Name of Sample	No. GenBank of nrITS	No. GenBank of *trn*L-*trn*F Region
*Schiedeella bajaverapacensis*	ON854135	ON862914

## Data Availability

Data supporting the reported results can be obtained from the corresponding author upon request.

## Appendix A

**Table A1:** List of the taxa that were used in the phylogenetic analyses with their accession numbers.

Taxon	Accession Number of ITS	Accession Number of trnL-trnF
*Aulosepalum nelsonii*	-------	MG582366.1
*Aulosepalum nelsonii* subsp. *obtusa*	AM884876.1	-------
*Aulosepalum riodelayensis*	AM884869.1	-------
*Beloglottis mexicana*	LT600852.1	LT600887.1
*Coccineorchis standleyi*	FN996949.1	-------
*Deiregyne albovaginata*	FN641870.1	FN641882.1
*Deiregyne densiflorus*	FN641874.1	FN641886.1
*Deiregyne diaphana* 1	KU752292.1	-------
*Deiregyne diaphana* 2	AJ539484.1	-------
*Deiregyne durangensis*	FN641867.1	FN641879.1
*Deiregyne eriophora*	FN641873.1	FN641885.1
*Deiregyne falcata*	FN641871.1	FN641883.1
*Deiregyne pseudopyramidalis*	FN641872.1	FN641884.1
*Deiregyne rhombilabia*	FN641869.1	FN641881.1
*Dichromanthus aurantiacus* 1	FN996957.1	FN996970.1
*Dichromanthus aurantiacus* 2	FN996956.1	FN996971.1
*Dichromanthus aurantiacus* 3	AJ539485.1	-------
*Dichromanthus cinnabarinus* 1	KU752293.1	-------
*Dichromanthus cinnabarinus* 2	AJ539486.1	-------
*Dichromanthus cinnabarinus* subsp. *cinnabarinus* 1	FN996952.1	FN996964.1
*Dichromanthus cinnabarinus* subsp. *cinnabarinus* 2	FN996951.1	FN996963.1
*Dichromanthus cinnabarinus* subsp. *galeottianus*	AM778176.1	FN996965.1
*Dichromanthus michuacanus* 1	FN996955.1	FN996969.1
*Dichromanthus michuacanus* 2	FN996954.1	FN996968.1
*Dichromanthus michuacanus* 3	FN996953.1	FN996967.1
*Dichromanthus michuacanus* 4	AM778177.1	FN996966.1
*Dichromanthus yucundaa*	FN996950.1	FN996962.1
*Greenwoodiella garayana 1*	LT600860.1	LT600894.1
*Greenwoodiella garayana* 2	LT600859.1	LT600893.1
*Greenwoodiella garayana* 3	LT600858.1	LT600895.1
*Greenwoodiella garayana* 4	LT600857.1	LT600892.1
*Greenwoodiella romeroana*	LT600861.1	LT600896.1
*Greenwoodiella wercklei* 1	LT600865.1	LT600901.1
*Greenwoodiella wercklei* 2	LT600864.1	LT600900.1
*Greenwoodiella wercklei* 3	-------	LT600899.1
*Kionophyton sawyeri*	LT600856.1	LT600891.1
*Kionophyton seminuda*	MF465022.1	MG582377.1
*Mesadenus chiangii*	MK309833.1	MK310230.1
*Mesadenus glaziovii* 1	MG460385.1	MG460417.1
*Mesadenus glaziovii* 2	MK309832.1	-------
*Mesadenus lucayanus* 1	MK309834.1	MK310231.1
*Mesadenus lucayanus* 2	KU752294.1	-------
*Mesadenus lucayanus* 3	AJ539488.1	-------
*Mesadenus polyanthus*	AM778175.1	LT600902.1
*Mesadenus tenuissimus* 1	MK309836.1	MK310233.1
*Mesadenus tenuissimus* 2	MK309835.1	MK310232.1
*Physogyne gonzalezii*	LT600855.1	LT600890.1
*Pseudogoodyera pseudogoodyeroides*	LT600854.1	LT600889.1
*Pseudogoodyera wrightii*	-------	MG582376.1
*Schiedeella affinis*	-------	LT600903.1
*Schiedeella crenulata*	FN641868.1	FN641880.1
*Schiedeella faucisanguinea*	AJ539496.1	-------
*Schiedeella llaveana* 1	KU752295.1	-------
*Schiedeella llaveana* 2	AJ539487.1	-------
*Schiedeella nagelii*	MK309837.1	MK310234.1
*Schiedeella* sp. 1	-------	LT600897.1
*Schiedeella* sp. 2	-------	LT600898.1
*Schiedeella tenella*	MK309838.1	MK310235.1
*Schiedeella williamsiana*	MK309839.1	MK310236.1
*Sotoa confusa*	FN641865.1	FN641876.1
*Spiranthes sinensis*	LT600853.1	LT600888.1
*Stenorrhynchos albidomaculatu*	FN996948.1	-------
*Stenorrhynchos millei*	FN996946.1	-------
*Stenorrhynchos speciosum*	FN996947.1	-------
